# The relationships between FLAIS, a novel insulin sensitivity index, and cardiovascular risk factors in a population-based study

**DOI:** 10.1186/s12933-022-01491-y

**Published:** 2022-04-19

**Authors:** Monika Karczewska-Kupczewska, Agnieszka Nikołajuk, Marcin Kondraciuk, Zofia Stachurska, Marlena Dubatówka, Anna Szpakowicz, Marek Strączkowski, Irina Kowalska, Karol Kamiński

**Affiliations:** 1 Department of Internal Medicine and Metabolic Diseases, Medical University of Białystok, M.C. Skłodowskiej 24a, 15-276 Białystok, Poland; 2 Department of Prophylaxis of Metabolic Diseases, Institute of Animal Reproduction and Food Research, Polish Academy of Sciences, Olsztyn, Poland; 3 Department of Population Medicine and Lifestyle Diseases Prevention, Medical University of Białystok, Białystok, Poland; 4 Department of Cardiology, Medical University of Białystok, Białystok, Poland

**Keywords:** Insulin resistance, Biomarker, Cardiovascular risk, Blood pressure, Lipids, Waist circumference

## Abstract

**Background:**

Insulin resistance is a risk factor for cardiovascular disease. Recently, we have developed a novel index, FLAIS (Fasting Laboratory Assessment of Insulin Sensitivity), which accurately reflects insulin sensitivity, measured with hyperinsulinemic-euglycemic clamp, in different groups of subjects. The aim of the present study was to assess the relationship of FLAIS with cardiovascular risk factors in a population-based study.

**Methods:**

The study group comprised 339 individuals from the ongoing Białystok Plus study, without previously known diabetes. Clinical examination, oral glucose tolerance test and the measurement of blood laboratory parameters were performed.

**Results:**

Prediabetes (impaired fasting glucose and/or impaired glucose tolerance) was diagnosed in 165 individuals whereas type 2 diabetes was diagnosed in 19 subjects. FLAIS was lower in individuals with prediabetes and diabetes in comparison with individuals with normal glucose tolerance. FLAIS was significantly related to waist circumference, systolic and diastolic blood pressure, triglycerides, HDL-cholesterol and LDL-cholesterol in the entire study group and in the subgroups with normal glucose tolerance and with prediabetes/diabetes. HOMA-IR, QUICKI and Matsuda index were not related to blood pressure and LDL-cholesterol in individuals with normal glucose tolerance. Majority of the adjusted models with FLAIS were characterized by better fit with the data in comparison with other indices for all cardiovascular risk factors except waist circumference.

**Conclusions:**

FLAIS represents useful index to assess the cluster of insulin resistance-associated cardiovascular risk factors in general population.

## Background

Insulin resistance is a risk factor for type 2 diabetes and cardiovascular disease (CVD) [1, 2]. Insulin resistance may accelerate atherogenesis and may contribute to CVD through numerous pathogenic pathways [1–3]. The causality of insulin resistance in coronary heart disease (CHD) development is supported by Mendelian randomization study, which demonstrated that single nucleotide polymorphisms influencing insulin resistance were associated with an increased risk of CHD [4]. In a mathematical modeling (“Archimedes model”) of simulated young (20–30 years) nondiabetic population, entered into a series of simulated clinical trials, insulin resistance was identified as the most important single cause of CHD. It was estimated that preventing insulin resistance in young adults would prevent 42% of myocardial infarctions [5].

Insulin resistance is associated with a cluster of CVD risk factors, such as central obesity, increased blood pressure, atherogenic dyslipidemia (increased triglycerides and decreased HDL-cholesterol), disturbances of glucose tolerance, which are together termed as metabolic syndrome [1, 6, 7]. These factors coexist together more often that may be explained by chance, and insulin resistance seems to play a crucial role in this clustering [1]. Insulin resistance may aggravate each of the components of metabolic syndrome, but may also independently promote inflammation and atherogenesis [1–3, 8]. It should also be noted that metabolic syndrome criteria are not effective in identifying insulin resistant individuals, and insulin resistant individuals not identified are also at increased CVD risk [9].

LDL-cholesterol is a well-established causal factor of atherosclerotic CVD [10]. Insulin decreases LDL-cholesterol through an increase in LDL receptor activity and LDL clearance [11]. In insulin resistant states, such as type 2 diabetes, LDL catabolism is decreased [12]. In the study of 1340 individuals with childhood or youth onset type 2 diabetes, the prevalence of dyslipidemia was 82% and high LDL-cholesterol was the most common lipid abnormality, present in 64.5% of individuals [13].

Current guidelines for CVD prevention in clinical practice include, together, with the factors describes above, conditions with the established link with insulin resistance, like increased body mass index (BMI) and type 2 diabetes, as well as interventions directed at improving insulin sensitivity, such as heathy diet and regular physical activity [14]. It is worth noting that even in nonobese nondiabetic individuals insulin resistance is predictor of CVD [15].

Thus, an early detection of insulin resistance may be important for CVD prevention. However, hyperinsulinemic-euglycemic clamp, “the gold standard” in measurement of insulin action in vivo [16], is laborious, costly and difficult to apply in everyday clinical practice. Indirect indices of insulin sensitivity/resistance utilize mainly fasting or oral glucose tolerance test (OGTT) glucose and insulin concentrations [17–19], however they usually display weaker accuracy in population without overt metabolic disturbances. The method of the measurement of insulin sensitivity may largely influence the results, as insulin sensitivity measured with hyerinsulinemic-euglycemic clamp, but not fasting insulin, was a predictor of CHD and stroke/transient ischemic attack [20, 21].

We developed a novel insulin sensitivity index, based on fasting laboratory parameters, called FLAIS (Fasting Laboratory Assessment of Insulin Sensitivity) [22], which accurately reflects insulin sensitivity, measured with hyperinsulinemic-euglcemic clamp, in different groups of subjects. This index utilizes red blood cell count (RBC), alanine aminotransferase (AlAt) activity, serum C-peptide, sex hormone-binding globulin (SHBG), adiponectin and insulin-like growth factor-binding protein 1 (IGFBP-1) concentrations. FLAIS displayed stronger correlations with clamp-derived insulin sensitivity than other indices studied [22]. Thus, we hypothesized that FLAIS may be associated with CVD risk factors.

The aim of the present study was to assess the relationship of FLAIS with CVD risk factors in a population-based study.

## Methods

### Study group

The study group comprised 339 individuals, 146 males and 193 females, without previously known diabetes. Participants aged 20–80 were randomly recruited from the city population database in order to reflect the general population as described previously [23]. All underwent clinical examination and appropriate laboratory tests [24]. Subject with active inflammation (CRP > 10 ng/mL) were excluded. OGTT was performed with the measurement of plasma glucose and serum insulin. Diabetes was diagnosed in patients with a glucose level at 2 h in OGTT ≥ 200 mg/dL. Prediabetes, impaired fasting glucose (IFG), and impaired glucose tolerance (IGT) were diagnosed as follows: IFG was diagnosed in patients with both fasting glucose levels 100–125 mg/dL and a glucose level at 2 h in OGTT < 140 mg/dL, and IGT was diagnosed in patients with a glucose level at 2 h in OGTT between 140 and 199 mg/dL. Glucose metabolism was considered normal if the fasting glucose level was < 100 mg/dL and the glucose level at 2 h in OGTT was < 140 mg/dL. Prediabetes (IFG and/or IGT) was diagnosed in 165 individuals whereas type 2 diabetes was diagnosed in 19 subjects. Due to the small number of subject with type 2 diabetes, individuals with prediabetes and individuals with newly diagnosed type 2 diabetes were pooled in the analyses (184 subjects, 96 males and 88 females). The remaining 155 subjects (50 males and 105 females) had normal glucose tolerance. There were no patients with unclear diabetic status in the study. In the entire study group, 14 individuals (4 with normal glucose tolerance and 10 with prediabetes/diabetes) had established CVD, including 7 with previous myocardial infarction (one person had also previous stroke), 4 with stable coronary heart disease, 3 with peripheral artery disease. Furthermore, in the entire study group, 219 individuals were not taking any medications influencing glucose metabolism or CVD risk factors, whereas 120 individuals were taking medications (most of them were taking more than one drug), including 31 receiving statins, 5—fibrates, 59—beta-blockers, 2—alpha-blockers, 22—acetylsalicylic acid, 43—angiotensin-converting enzyme inhibitors, 21—angiotensin receptor blockers, 28—calcium channel blockers, 22—diuretics, 22—levothyroxine and 3—steroids. The study protocol was approved by the local ethics committee of the Medical University of Białystok, Poland, Approval no: R-I-002/108/2016. A written informed consent was obtained from all individuals before their participation in the study.

### Laboratory analyses

Plasma glucose, serum insulin, lipids were measured with standard laboratory procedures as described previously [23]. We also analyzed indirect indices of insulin sensitivity/resistance based on fasting or OGTT plasma glucose and serum insulin concentrations, homeostasis model assessment-insulin resistance (HOMA-IR) [17], quantitative insulin sensitivity check index [18] and Matsuda index (available in 337 subjects because no insulin measurements from OGTT were available in 2 subjects) [19].

Blood morphology, serum aspartate aminotransferase (AspAt) and AlAt activities were measured as previously described [22]. For the remaining measurements, serum and plasma samples were stored at − 80 °C until analyses. Serum C-peptide, SHBG, adiponectin and IGFBP-1 concentrations were measured as previously described [22].

### Calculation of FLAIS

FLAIS was calculated as described previously [22]:

$${\text{FLAIS}} =\,11.5847 - {\text{red blood cells }}({\text{RBC}}) \times 0.9622 - {\text{AlAt}} \times 0.0308 - {\text{C-peptide}} \times 1.0718 + {\text{SHBG}} \times 0.0239 + {\text{Adiponectin}} \times 0.0466+{\text{IGFBP-1}} \times 0.1206.$$

### Statistical analysis

The statistical analysis was performed using STATISTICA 13.5 software (StatSoft Poland, Kraków). The descriptive data are presented as mean ± SD. Shapiro–Wilk test was used to asses normal distribution. Variables, which did not have normal distribution (fasting and post-OGTT insulin, triglycerides, HOMA-IR, Matsuda index, AlAt, SHBG, adiponectin and IGFBP-1) were log-transformed prior to an analyses. For the purpose of the data presentation, absolute values are shown in “Results” section. The differences between the groups with and without disturbance of glucose tolerance were estimated with the unpaired Student’s t test. The relationships between variables were studied with Pearson product moment correlation analysis and with multiple regression analysis. The differences between 2 correlation coefficients were analyzed with Fisher z transformation and two-tailed Fisher z test. The level of significance was accepted at p < 0.05.

Different multiple regression analysis models estimating the relationships of FLAIS and other indices of insulin sensitivity/resistance with CVD risk factors adjusted for age, sex, taking medications (or not) and glucose tolerance status, were compared using Akaike Information Criterion (AIC) with GraphPad Prism version 9.3.1 (GraphPad Software, San Diego, CA, USA). AIC determines which model out of 2 models analyzed better fits the data. The better model is characterized by lower AIC. Analysis of AIC indicates also the probability that the preferred model is correct.

## Results

### The characteristics of the study group

Subjects with prediabetes/diabetes had higher BMI, waist circumference, systolic and diastolic blood pressure, fasting and post-OGTT glucose and insulin, HOMA-IR and lower QUICKI and Matsuda index in comparison with individuals with normal glucose tolerance (all p < 0.001) (Table 1). Triglycerides (p < 0.001) and LDL-cholesterol (p = 0.014) were higher and HDL-cholesterol (p = 0.014) was lower in subjects with prediabetes/diabetes (Table 1).

**Table 1:** Clinical characteristics of the groups with normal glucose tolerance and with prediabetes/type 2 diabetes

	Normal glucose tolerance (n = 155)	Prediabetes/diabetes (n = 184)
Age (years)	42.33 ± 14.23	51.61 ± 14.19*
Sex (M/F)	50/105	96/88*
Systolic BP (mmHg)	117.42 ± 14.42	128.88 ± 18.14*
Diastolic BP (mmHg)	78.91 ± 8.88	83.96 ± 11.17*
BMI (kg/m^2^)	24.79 ± 4.14	27.96 ± 4.59*
Waist circumference (cm)	81.12 ± 11.25	91.06 ± 12.96*
Fasting plasma glucose (mg/dL)	93.24 ± 4.53	106.16 ± 10.38*
Post-OGTT plasma glucose (mg/dL)	107.29 ± 18.92	141.25 ± 41.93*
Fasting serum insulin (μIU/mL)	9.71 ± 5.03	14.49 ± 9.19*
Post-OGTT serum insulin (μIU/mL)	43.13 ± 28.42	82.92 ± 91.91*
Cholesterol (mg/dL)	182.36 ± 37.64	190.34 ± 38.14
Triglycerides (mg/dL)	96.28 ± 60.19	118.01 ± 72.89*
HDL-cholesterol (mg/dL)	63.05 ± 16.60	58.93 ± 14.17*
LDL-cholesterol (mg/dL)	117.84 ± 32.37	127.10 ± 35.76*
HOMA-IR	2.25 ± 1.20	3.88 ± 2.82*
QUICKI	0.35 ± 0.02	0.32 ± 0.03*
Matsuda index	6.21 ± 2.71	3.81 ± 2.28*
RBC (mln/μL)	4.71 ± 0.46	4.83 ± 0.40*
AlAt (U/L)	19.89 ± 11.67	25.06 ± 14.83*
C-peptide (pmol/mL)	0.71 ± 0.22	0.92 ± 0.38*
SHBG (nmol/L)	38.55 ± 25.79	30.44 ± 17.92*
Adiponectin (μg/mL)	16.18 ± 9.29	10.05 ± 7.29*
IGFBP-1 (ng/mL)	3.18 ± 2.70	2.12 ± 1.72*
FLAIS	7.74 ± 1.59	6.64 ± 1.41*

*p < 0.05 vs normal glucose tolerance

All parameters forming the FLAIS index differed between the group with normal glucose tolerance and the group with prediabetes/diabetes. RBC (p = 0.012), AlAt, C-peptide (both p < 0.001) were higher whereas SHBG (p = 0.002), adiponectin and IGFBP-1 (both p < 0.001) were lower in individuals with prediabetes/diabetes in comparison with individuals with normal glucose tolerance (Table 1). FLAIS was lower in the group with prediabetes/diabetes in comparison with the group with normal glucose tolerance (p < 0.001) (Table 1).

### Correlations between FLAIS, other indices of insulin sensitivity/resistance and cardiovascular risk factors

FLAIS was related to systolic and diastolic blood pressure, waist circumference, log triglycerides, HDL-cholesterol and LDL-cholesterol in the entire study group (Table 2). Correlations of FLAIS with CVD risk factors had coefficients higher or comparable as those observed for other indices of insulin sensitivity/resistance. We were able to detect statistically significant differences for the correlations of FLAIS with systolic blood pressure and HDL-cholesterol in comparison with the correlations of other indices with these parameters (systolic blood pressure, HOMA-IR p = 0.033; QUICKI, p = 0.024; HDL-cholesterol, HOMA-IR and QUICKI, p = 0.036; Matsuda p = 0.0007; p values for the comparison between 2 correlation coefficients). The correlation coefficients for FLAIS were higher than for the parameters forming FLAIS analyzed separately, except AlAt and LDL-cholesterol (Table 3). Exclusion of subjects with established CVD or receiving any treatment did not change the results (data not shown).

**Table 2:** Correlations between indirect indices of insulin sensitivity/resistance and cardiovascular risk factors in the entire study group (n = 339)

	FLAIS		HOMA-IR		QUICKI		Matsuda index	
	r	p	r	p	r	p	r	p
Systolic blood pressure	− 0.37	< 0.001	0.22	< 0.001	− 0.21	< 0.001	− 0.26	< 0.001
Diastolic blood pressure	− 0.33	< 0.001	0.32	< 0.001	− 0.31	< 0.001	− 0.32	< 0.001
Waist	− 0.64	< 0.001	0.63	< 0.001	− 0.61	< 0.001	− 0.60	< 0.001
Triglycerides	− 0.42	< 0.001	0.41	< 0.001	− 0.40	< 0.001	− 0.44	< 0.001
HDL-cholesterol	0.51	< 0.001	− 0.37	< 0.001	0.37	< 0.001	0.34	< 0.001
LDL-cholesterol	− 0.21	< 0.001	0.21	<0.001	− 0.21	< 0.001	− 0.19	< 0.001

**Table 3:** Correlations between parameters forming FLAIS index and cardiovascular risk factors in the entire study group (n = 339)

	RBC		AlAt		C-peptide		SHBG		Adiponectin		IGFBP-1	
	r	p	r	p	r	p	r	p	r	p	r	p
Systolic blood pressure	0.25	< 0.001	0.30	< 0.001	0.26	< 0.001	− 0.28	< 0.001	− 0.20	< 0.001	− 0.28	< 0.001
Diastolic blood pressure	0.24	< 0.001	0.27	< 0.001	0.26	< 0.001	− 0.22	< 0.001	− 0.16	0.003	− 0.29	< 0.001
Waist	0.40	< 0.001	0.49	< 0.001	0.55	< 0.001	− 0.45	< 0.001	− 0.37	< 0.001	− 0.51	< 0.001
Triglycerides	0.25	< 0.001	0.39	< 0.001	0.39	< 0.001	− 0.31	< 0.001	− 0.33	< 0.001	− 0.25	< 0.001
HDL-cholesterol	− 0.37	< 0.001	− 0.29	< 0.001	− 0.29	< 0.001	0.44	< 0.001	0.44	< 0.001	0.36	< 0.001
LDL-cholesterol	0.15	0.006	0.23	< 0.001	0.12	0.024	− 0.20	< 0.001	0.03	0.60	− 0.15	0.007

We also analyzed correlations of FLAIS with cardiovascular risk factors n the groups of subjects with normal glucose tolerance and with prediabetes/diabetes separately. In the group with normal glucose tolerance, FLAIS was related to systolic and diastolic blood pressure, waist circumference, triglycerides, HDL-cholesterol and LDL-cholesterol. In this group, none of other indices analyzed was related to systolic blood pressure, diastolic blood pressure and LDL-cholesterol (Table 4).

**Table 4:** Correlations between indirect indices of insulin sensitivity/resistance and cardiovascular risk factors in the group with normal glucose tolerance (n = 155)

	FLAIS		HOMA-IR		QUICKI		Matsuda index	
	r	p	r	p	r	p	r	p
Systolic blood pressure	− 0.35	< 0.001	0.02	0.77	− 0.01	0.95	− 0.04	0.63
Diastolic blood pressure	− 0.22	0.006	0.14	0.075	− 0.14	0.16	− 0.15	0.057
Waist	− 0.60	< 0.001	0.52	< 0.001	− 0.49	< 0.001	− 0.43	< 0.001
Triglycerides	− 0.26	0.001	0.22	0.006	− 0.21	0.01	− 0.28	< 0.001
HDL-cholesterol	0.52	< 0.001	− 0.39	< 0.001	0.38	< 0.001	0.34	< 0.001
LDL-cholesterol	− 0.20	0.012	0.14	0.087	− 0.14	0.079	− 0.14	0.082

In the group with prediabetes/diabetes, FLAIS was also related to systolic and diastolic blood pressure, waist circumference, log triglycerides, HDL-cholesterol and LDL-cholesterol. Correlations of FLAIS with systolic and diastolic blood pressure and HDL-cholesterol had slightly higher correlation coefficients than those observed for other indices whereas those for other parameters analyzed they were comparable (Table 5).

**Table 5:** Correlations between indirect indices of insulin sensitivity/resistance and cardiovascular risk factors in the group with prediabetes/diabetes (n = 184)

	FLAIS		HOMA-IR		QUICKI		Matsuda index	
	r	p	R	p	r	p	r	p
Systolic blood pressure	− 0.25	0.001	0.17	0.023	− 0.15	0.044	− 0.19	0.01
Diastolic blood pressure	− 0.32	< 0.001	0.31	< 0.001	− 0.30	< 0.001	− 0.30	< 0.001
Waist	− 0.57	< 0.001	0.58	< 0.001	− 0.57	< 0.001	− 0.57	< 0.001
Triglycerides	− 0.49	< 0.001	0.46	< 0.001	− 0.47	< 0.001	− 0.48	< 0.001
HDL-cholesterol	0.48	< 0.001	− 0.32	< 0.001	0.32	< 0.001	0.31	< 0.001
LDL-cholesterol	− 0.16	0.03	0.19	0.01	− 0.19	0.01	− 0.16	0.028

### Multiple regression analysis

After adjustment for age, sex, taking (or not) medications, and for glucose tolerance status (normal glucose tolerance vs prediabetes/diabetes), FLAIS was still significantly related to all analyzed cardiovascular risk factors (Table 6). Regarding other indices, none of them was significantly related to systolic blood pressure in the adjusted models. In most cases, R^2^ of the models with FLAIS was higher than for HOMA-IR, QUICKI and Matsuda index for all cardiovascular risk factors except waist circumference.

**Table 6:** Multiple regression analysis models for the correlations of FLAIS and other indices with CVD risk factors

	Systolic blood pressure		Diastolic blood pressure		Waist circumference		Triglycerides		HDL-cholesterol		LDL-cholesterol	
	β	p	β	p	β	p	β	p	β	p	β	p
Model 1												
Age	**0.32**	**< 0.001**	**0.18**	**0.006**	**0.27**	**< 0.001**	**0.25**	**< 0.001**	0.01	0.81	**0.24**	**0.0003**
Sex (M/F)	− **0.28**	**<** **0.001**	− 0.01	0.82	−** 0.18**	**0.0002**	− 0.01	0.96	**0.24**	**<** **0.001**	0.08	0.23
Medication (no/yes)	− 0.06	0.32	− 0.08	0.25	0.21	0.18	0.04	0.50	− **0.11**	**0.044**	− 0.06	0.39
Normal glucose tolerance (yes/no)	**0.11**	**0.046**	0.09	0.13	0.05	0.24	− 0.04	0.45	0.08	0.15	− 0.01	0.92
FLAIS	− **0.19**	**0.004**	− **0.31**	**<** **0.001**	− **0.52**	**<** **0.001**	− **0.44**	**<** **0.001**	**0.38**	**<** **0.001**	− **0.28**	**<** **0.001**
R^2^ of the model	0.29		0.15		0.53		0.24		0.31		0.09	
Corrected R^2^	0.28		0.13		0.52		0.23		0.29		0.08	
AIC	1790		1530		1457		− 1106		1746		2381	
Model 2												
Age	**0.29**	**<** **0.001**	**0.14**	**0.03**	**0.20**	**<** **0.001**	**0.19**	**0.002**	0.06	0.26	**0.20**	**0.003**
Sex (M/F)	− **0.38**	**<** **0.001**	− **0.15**	**0.005**	− **0.41**	**<** **0.001**	− **0.20**	**<** **0.001**	**0.41**	**<** **0.001**	− 0.05	0.32
Medication (no/yes)	− 0.04	0.52	− 0.06	0.32	0.08	0.08	0.06	0.30	− **0.13**	**0.017**	− 0.03	0.59
Normal glucose tolerance (yes/no)	**0.13**	**0.021**	0.07	0.21	− 0.01	0.91	− 0.06	0.29	0.09	0.09	0.001	0.99
HOMA-IR	0.08	0.15	**0.25**	**<** **0.001**	**0.51**	**<** **0.001**	**0.36**	**<** **0.001**	− **0.30**	**<** **0.001**	**0.18**	**0.003**
R^2^ of the model	0.28		0.14		0.59		0.24		0.30		0.07	
Corrected R^2^	0.27		0.13		0.59		0.23		0.29		0.06	
AIC	1796		1531		1407		− 1104		1748		2388	
Δ AIC	− 6.52		− 0.501		49.26		− 2.214		− 2.252		− 7.03	
The preferred model (comparison with Model 1)	Model 1 (FLAIS)		Model 1 (FLAIS)		Model 2 (HOMA-IR)		Model 1 (FLAIS)		Model 1 (FLAIS)		Model 1 (FLAIS)	
The probability that the referred model is correct	96.3%		56.23%		99.99%		75.16%		75.51%		97.11%	
Model 3												
Age	**0.29**	**<** **0.001**	**0.14**	**0.032**	**0.20**	**<** **0.001**	**0.19**	**0.002**	0.06	0.26	**0.20**	**0.003**
Sex (M/F)	− **0.38**	**<** **0.001**	− **0.16**	**0.004**	− **0.42**	**<** **0.001**	− **0.21**	**<** **0.001**	**0.41**	**<** **0.001**	− 0.06	0.31
Medication (no/yes)	− 0.03	0.55	− 0.06	0.36	0.09	0.053	0.07	0.26	− **0.14**	**0.014**	− 0.03	0.61
Normal glucose tolerance (yes/no)	**0.13**	**0.017**	0.08	0.19	− 0.01	0.91	− 0.06	0.29	0.09	0.078	− 0.01	0.97
QUICKI	− 0.06	0.24	− **0.24**	**<** **0.001**	− **0.50**	**<** **0.001**	− **0.35**	**<** **0.001**	**0.31**	**<** **0.001**	− **0.18**	**0.002**
R^2^ of the model	0.27		0.14		0.58		0.24		0.30		0.08	
Corrected R^2^	0.26		0.13		0.58		0.22		0.29		0.06	
AIC	1797		1533		1413		− 1103		1747		2387	
Δ AIC	− 7.253		− 2.72		43.23		− 3.237		− 1.127		− 6.554	
The preferred model (comparison with Model 1)	Model 1 (FLAIS)		Model 1 (FLAIS)		Model 3 (QUICKI)		Model 1 (FLAIS)		Model 1 (FLAIS)		Model 1 (FLAIS)	
The probability that the referred model is correct	97.41%		79.58%		99.99%		83.46%		63.73%		96.36%	
Model 4												
Age	**0.29**	**<** **0.001**	0.12	0.069	**0.16**	**<** **0.001**	**0.16**	**0.006**	0.09	0.11	**0.19**	**0.003**
Sex (M/F)	− **0.38**	**<** **0.001**	− **0.16**	**0.003**	− **0.43**	**<** **0.001**	− **0.22**	**<** **0.001**	**0.42**	**<** **0.001**	− 0.08	0.16
Medication (no/yes)	− 0.04	0.50	− 0.06	0.37	**0.09**	**0.046**	0.06	0.29	− **0.14**	**0.011**	− 0.04	0.52
Normal glucose tolerance (yes/no)	**0.11**	**0.045**	0.06	0.29	− 0.01	0.77	− 0.09	0.099	0.10	0.059	− 0.01	0.96
Matsuda index	− 0.10	0.06	− **0.26**	**<** **0.001**	− **0.49**	**<** **0.001**	− **0.40**	**<** **0.001**	**0.31**	**<** **0.001**	− **0.15**	**0.012**
R^2^ of the model	0.28		0.14		0.56		0.26		0.30		0.07	
Corrected R^2^	0.27		0.13		0.56		0.25		0.29		0.06	
AIC	1786 (vs 1781)		1524 (vs 1523)		1422 (vs 1450)		− 1106 (vs − 1099)		1739 (vs 1736)		2367 (vs 2358)	
Δ AIC	− 5.011		− 1.282		27.59		7.65		− 3.304		− 9.047	
The preferred model (comparison with Model 1)	Model 1 (FLAIS)		Model 1 (FLAIS)		Model 4 (Matsuda)		Model 4 (Matsuda)		Model 1 (FLAIS)		Model 1 (FLAIS)	
The probability that the referred model is correct	92.45%		65.5%		99.99%		97.86%		83.91%		98.93%	

Significant beta coefficients are indicated in bold. AIC, Akaike Information Criterion. The lower AIC, the better fit of the model. Δ AIC was calculated as AIC model 1—AIC model 2, 3 or 4, i.e. the negative values indicate that the preferred model is model 1 (with FLAIS)

Matsuda index was calculated in 337, therefore, slightly different AIC from Model 1 was taken for comparisons and ΔAIC calculation

The direct comparison between adjusted models containing FLAIS with adjusted models containing other indices using AIC is presented in Table 6. In most cases, models with FLAIS were preferred over the models with HOMA-IR, QUICKI or Matsuda index for all cardiovascular risk factors analyzed. The exceptions were: waist circumference (all indices), triglycerides and the model with Matsuda index. For systolic blood pressure the probability that the model with FLAIS is the preferred model was over 90% and for LDL-cholesterol it was over 95% (Table 6). For triglycerides (except the model with Matsuda index) and HDL-cholesterol the probability that the model with FLAIS is the preferred model ranged from 63.7 to 83.9%, whereas for diastolic blood pressure the range of the probability was from 56.2 to 79.6% in comparison with the models with 3 other indices analyzed (Table 6).

## Discussion

In the present study, we demonstrated that novel insulin sensitivity index, FLAIS, is significantly associated with CVD risk factors in population-based cohort study and thus can be applied in general population, both in individuals with normal glucose tolerance and with individuals with prediabetes/diabetes. Furthermore, HOMA-IR, QUICKI and Matsuda index were not related to blood pressure and LDL-cholesterol in individuals with normal glucose tolerance. Majority of the adjusted models with FLAIS were characterized by better fit with the data in comparison with other indices for all risk factors except waist circumference.

 Identification of insulin resistance in population without disturbances in glucose tolerance may be important for CVD prevention. In our study, FLAIS was significantly related to waist circumference, systolic and diastolic blood pressure, triglycerides, HDL-cholesterol and LDL-cholesterol not only in the entire study group, but also in the subgroup with normal glucose tolerance. Importantly, none of other indices analyzed was significantly related to all CVD risk factors in the group with normal glucose tolerance. In the aforementioned study with insulin sensitivity as a negative predictor of CHD, the population was nondiabetic and approx. 80% had normal glucose tolerance at the baseline of 10-year observation [20]. In the study of 295 adolescents, insulin resistance assessed with hyperinsulinemic-euglycemic clamp was associated with CVD risk factors, systolic blood pressure, triglycerides, HDL-cholesterol and fasting insulin, and interacted with obesity in these associations [25]. It is well established that insulin resistance is related to blood pressure [26] and that subjects with hypertension demonstrate a decreased insulin sensitivity even at the early stage of the disease [27]. In this context, the relationships of FLAIS with systolic and diastolic blood pressure in the group with normal glucose tolerance provide a possible advantage of FLAIS over other indices based on glucose and insulin measurements in assessing insulin resistance-associated CVD risk in a population with normal glucose tolerance.

It is worth to underline that FLAIS, but not other indices, was related to LDL-cholesterol in the group with normal glucose tolerance. LDL-cholesterol is not a component of metabolic syndrome. However, as already mentioned, it may also be related to insulin resistance and it may also act synergistically with the components of metabolic syndrome to accelerate atherogenesis [28].

FLAIS was lower in individuals with prediabetes/diabetes and was also related to all analyzed CVD risk factors in this group. Prediabetes itself is CVD risk factor [29]. It increased the risk of unrecognized myocardial infarction in comparison to normal glucose tolerance in population without CVD at baseline [30]. Prediabetes is also associated with an increased values of the individual components of metabolic syndrome [31], as we also observed in our study. It is important to note that FLAIS was related to all CVD risk factors also in the group with prediabetes/diabetes and the correlations coefficients were slightly higher or comparable to the correlations observed for the indices based on blood glucose and insulin concentrations. One may suppose that FLAIS may accurately reflect the cluster of CVD risk factors associated with insulin resistance also in prediabetes and diabetes. It should be noted that in the adjusted models, FLAIS, but not the presence of prediabetes or diabetes, was a significant predictor of all analyzed cardiovascular risk factors. These data additionally indicate the importance of focusing on insulin resistance in the prevention of CVD.

The correlation coefficients between FLAIS and CVD risk factors were also higher than those observed for individual parameters forming FLAIS. In previous studies, increased RBC was a predictor of CVD events in 6-year follow-up [32]. Serum AlAt activity was associated with most CVD risk factors [33]. Serum C-peptide was identified as a predictor of CVD and overall death in nondiabetic adults, better than glucose and/or insulin-derived measures [34]. Higher serum SHBG was associated with a more favorable cardiometabolic risk profile [35]. Adiponectin is an adipokine with insulin-sensitizing, anti-inflammatory and antiatherogenic properties, which is inversely related to CVD risk factors [36, 37]. IGFBP- was associated with CVD events in an analysis of 3523 Framingham Heart Study participants [38]. These results are in agreement with our data showing significant correlations between parameters forming FLAIS and CVD risk factors in almost all situations (except correlations of adiponectin with LDL-cholesterol). However, the correlation coefficients of FLAIS with CVD risk factors were higher than those observed for individual parameters of FLAIS formula. Thus, similarly to the correlation between FLAIS and clamp-derived insulin sensitivity, the strength of FLAIS comes from the unique combination of the individual parameters and from balancing the contribution of each variable to the final equation.

It should be noted that Białystok Plus study is a population-based study and the individuals recruited for the study had different medical conditions and received different treatment. Our results give a support for the usefulness of FLAIS in the studies of general population. The usefulness of FLAIS as a predictor of CVD should be studies further in prospective studies.

## Conclusions

FLAIS represents useful index to assess the cluster of insulin resistance-associated CVD risk factors in general population.

## Data Availability

The datasets analysed in the current study are available from the corresponding author on reasonable request.

## Abbreviations

CVD: Cardiovascular disease
CHD: Coronary heart disease
BMI: Body mass index
OGTT: Oral glucose tolerance test
FLAIS: Fasting laboratory assessment of insulin sensitivity
RBC: Red blood cells
AlAt: Alanine aminotransferase
SHBG: Sex hormone-binding globulin
IGFBP-1: Insulin-like growth factor-binding protein 1
IFG: Impaired fasting glucose
IGT: Impaired glucose tolerance
HOMA-IR: Homeostasis model assessment-insulin resistance
QUICKI: Quantitative insulin sensitivity check index
